# Ex-vivo RNA expression analysis of vaccine candidate genes in COPD sputum samples

**DOI:** 10.1186/s12931-023-02525-z

**Published:** 2023-10-05

**Authors:** Cecilia Brettoni, Alessandro Muzzi, Simona Rondini, Vincent Weynants, Silvia Rossi Paccani

**Affiliations:** 1 GSK, Via Fiorentina 1, Siena, Italy; 2 GSK, Rixensart, Belgium

**Keywords:** Acute exacerbation, Chronic obstructive pulmonary disease, Droplet digital RT-PCR, *Moraxella catarrhalis*, Non-typeable *Haemophilus influenzae*, RNA expression, Sputum

## Abstract

**Background:**

Chronic obstructive pulmonary disease (COPD) is a lung disease characterised by airflow-limiting inflammation and mucus production. Acute exacerbations are a major cause of COPD-related morbidity and mortality and are mostly associated with bacterial or viral infections. A vaccine targeting non-typeable *Haemophilus influenzae* (NTHi) and *Moraxella catarrhalis* (Mcat), the main bacteria associated with exacerbations, was tested in a Phase 2 trial. We assessed “ex-vivo” expression of vaccine candidate and housekeeping genes *pd*, *pe*, *pilA*, *gapA*, *ompP6* of NTHi, and *uspA2*, *parE*, *polA* of Mcat in sputum samples of COPD patients and determined whether expression of the vaccine candidate genes *pd*, *pe*, *pilA* (NTHi) and *uspA2* (Mcat) differed between stable and exacerbation samples.

**Methods:**

A single-centre, prospective, observational cohort study was conducted where 123 COPD patients were seen on enrolment, followed monthly for 2 years, and reviewed after onset of acute exacerbations. We selected 69 patients with sputum samples positive for NTHi or Mcat by PCR during at least one stable and one exacerbation visit. mRNA was isolated from the sputum, and expression of NTHi and Mcat genes was analysed with RT-PCR. Statistical analyses compared mRNA concentrations between stable and exacerbation samples and in relationship to COPD severity and exacerbation frequency.

**Results:**

The vaccine candidate genes were variably expressed in sputum samples, suggesting they are expressed in the lung. Absolute and relative expression of all NTHi vaccine candidate genes and Mcat *uspA2* were similar between exacerbation and stable samples. Expression of *pd* and *pilA* was slightly associated with the number of exacerbations in the year before enrolment, and *uspA2* with the disease severity status at enrolment.

**Conclusions:**

The NTHi-Mcat vaccine candidate genes were expressed in sputum samples, and each gene had a specific level of expression. No statistically significant differences in gene expression were detectable between stable and exacerbation samples. However, the history of COPD exacerbations was slightly associated with the expression of *pd*, *pilA* and *uspA2*.

*Trial registration* NCT01360398 (https://www.clinicaltrials.gov)

**Graphical abstract:**

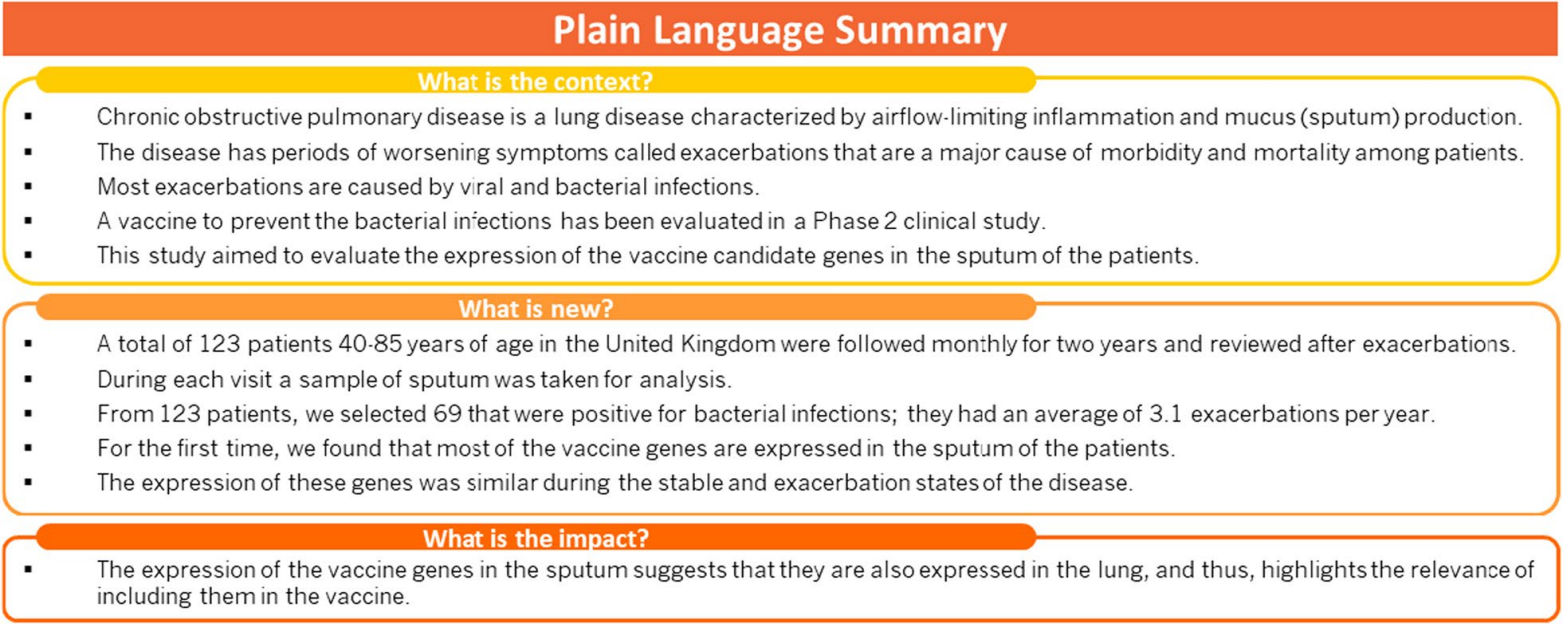

**Supplementary Information:**

The online version contains supplementary material available at 10.1186/s12931-023-02525-z.

## Background

Chronic obstructive pulmonary disease (COPD) is a lung disease characterised by airflow-limiting inflammation and mucus production. Short periods of worsening symptoms, called exacerbations, progressively aggravate the disease [1]. COPD is the most common chronic respiratory illness in older adults and the third leading cause of mortality worldwide, responsible for about 6% of all global deaths in 2019 [2]. COPD substantially impacts quality of life and imposes a heavy socioeconomic burden [3]. The total annual cost of COPD in Europe was €141.4 billion in 2011 [3].

Acute Exacerbation of COPD (AECOPD) is a major cause of COPD-related morbidity and mortality and accounts for a large proportion of COPD’s economic burden [3–7]. AECOPD is triggered by various causes, such as pneumonia, pulmonary embolism, or inhalation of irritants [1], but is mostly associated with viral and bacterial infections. Bacterial infections alone are associated with about 50% of exacerbations, and non-typeable *Haemophilus influenzae* (NTHi), *Streptococcus pneumoniae*, and *Moraxella catarrhalis* (Mcat) are the bacteria most often identified [8].

One of the strategies to prevent exacerbations would be vaccinating COPD patients against the most common pathogens causing AECOPD. Vaccines against *Streptococcus pneumoniae* already exist as they were developed to prevent streptococcal pneumonia and meningitis. No vaccine exists against NTHi and Mcat. Since NTHi lacks a polysaccharide capsule, vaccine development efforts have been concentrated on identifying NTHi surface proteins that are immunogenic and highly conserved within the species.

A candidate vaccine against NTHi and Mcat composed of three NTHi antigens (type IV pilin protein [PilA], protein E [PE], and protein D [PD]) and one Mcat antigen (ubiquitous surface protein A2 [UspA2]) has been developed and tested in a Phase 2 efficacy trial [9]. PilA is a pilus subunit involved in biofilm formation and mobility whereas PE and PD are involved in serum resistance and adherence. UspaA2 mediates both bacterial adherence and complement-mediated killing resistance through the binding of extracellular matrix proteins, including vitronectin, collagen, and laminin. To improve our understanding of the role of infections in AECOPD, the prospective longitudinal epidemiological study Acute Exacerbation and Respiratory InfectionS in COPD (AERIS) was conducted [10]. With the current study, we aimed to build upon the findings from AERIS by assessing the expression of NTHi-Mcat vaccine candidate genes in sputum of COPD patients who participated in the AERIS study. Sputum samples were processed to limit their possible alterations and to allow RNA quantification in conditions mirroring in-vivo situation, and referred to as “ex-vivo” in this manuscript. Additionally, we determined whether expression of such genes differed between samples at stable routine visits (ST) and exacerbation visits (EX), in relation to COPD severity and AECOPD frequency.

## Methods

### Study design

A single-centre, prospective, observational cohort study was conducted at University Hospital Southampton, UK. The clinical study was registered with the identification number NCT01360398 [11]. Participants were recruited from June 2011 to June 2012, and the study was performed between 30 June 2011 and 27 June 2014. Participants were seen at the enrolment visit and then followed monthly for 2 years. Each subject was visited within 72 h of AECOPD onset.

Severity of COPD condition was classified by forced expiratory volume in one second (FEV1) measurements in mild, moderate, severe or very severe, according to the Global Initiative for Chronic Obstructive Lung Disease (GOLD) staging at each visit [12].

### Participants

Participants 40–85 years were included according to the inclusion and exclusion criteria described for the AERIS study [10].

### Sample collection and selection

All procedures for sputum sampling and pathogen detection were described previously [10, 12]. Briefly, sputum samples were collected at study entry, monthly, and within 72 h of exacerbation by spontaneous expectoration or by induction and were processed with standard methods to prevent potential changes such as RNA degradation. The processed samples are referred to as “ex-vivo” samples. Specific pathogens were identified in the ex-vivo sputum samples with conventional culture techniques and quantitative DNA PCR [13]. Bacterial DNA detection thresholds were 2000 and 15,000 copies/ml for NTHi and Mcat, respectively [13].

This investigation aimed to measure, in the ex-vivo sputum samples of COPD patients, the RNA expression levels of specific NTHi and Mcat antigens considered in the NTHi-Mcat vaccine developed by GSK. The expression levels of the vaccine candidate genes were normalised to the ones of housekeeping genes *gapA*, *ompP6* of NTHi, or to their average combination as described in [14], and *parE*, *polA* of Mcat, or to their average combination [14]. For this purpose, patients were selected who had sputum samples that were positive for NTHi or Mcat by PCR during ≥ 1 EX and ≥ 1 ST. If samples from multiple visits were positive for the same pathogen, from the same patient and condition, one sputum sample was randomly selected for the RNA expression investigation.

### RNA extraction from sputum

The complete procedure of sputum preparation is described in the study protocol [10]. Total RNA was extracted from the sputum samples using TRIzol (Invitrogen), adding gDNA eliminator solution (Qiagen) to reduce gDNA contamination in the aqueous phase. Samples were purified with an RNeasy mini kit (Qiagen).

### RT-PCR reactions

Droplet digital RT-PCR (RT-ddPCR) reactions were performed using the One-Step RT-ddPCR Advanced Kit for Probes (Bio-Rad). Additional file 1: Table S1 lists primer sequences and annealing temperatures for the NTHi *pd*, *pe*, and *pilA* genes, which encode NTHi-Mcat vaccine antigens PD, PE, and PilA; the NTHi housekeeping genes *gapA* (encoding Gapdh) and *ompP6*; the Mcat gene *uspA2*, which encodes NTHi-Mcat vaccine antigen UspA2; and Mcat housekeeping genes *parE* and *polA*. Primers were purchased from Sigma-Aldrich. Droplets were generated by adding 20 μl PCR-mix and 70 μl Droplet Generation Oil for Probes (Bio-Rad) to the wells in a Droplet Generator DG8 Cartridge (Bio-Rad) before covering the cartridges with DG8 Gaskets (Bio-Rad) and placing them in a QX100 Droplet Generator (Bio-Rad). Afterward, the droplets were transferred to 96-well PCR plates (Eppendorf) that were sealed and placed in a C1000 Touch Thermal Cycler (Bio-Rad) for RT-ddPCR amplification. During the set-up of the procedure, various negative controls (e.g., samples without reverse transcriptase) were included. Data analysis and acquisition were performed with QuantaSoft (Bio-Rad). As per Bio-Rad protocols, samples with counts < 10,000 were rejected and considered as not measurable during the analysis. Droplet counts ≥ 10,000 and reporting ‘No call’ as an automatically estimated concentration were considered as technically measurable but below the limit of detection of the instrument and corrected to the value of 0.1 copies/μl.

### Statistical analyses

Absolute and relative concentrations of the RT-PCR products of the NTHi and Mcat antigens were compared between stable and exacerbations by Wilcoxon rank sum test (for group comparison) and signed rank test (for paired comparison by patient). Absolute and relative concentrations were also analysed by a rank linear regression statistical model or a rank-based analysis of variance to evaluate associations between gene expression and other variables recorded during the study, such as COPD severity (by GOLD definition at enrolment or at each visit), a subject’s total number of exacerbations during the study, and NTHi and Mcat DNA PCR concentrations measured at single visits or as average of all visits. The statistical models were built by grouping samples by visit type (ST or EX) and are described in Additional file 1: Text. All analyses were performed using R software version 4.2.1. Wilcoxon tests were applied by *wilcox.test* function of the stats package, and regression models were applied by *rfit* and *raov* functions of the Rfit package. P-values < 0.05 were considered statistically significant.

## Results

### Patients and samples

Initially, 127 patients were enrolled. COPD severity, based on FEV1, was 44.9% moderate, 40.2% severe, and 15.0% very severe. Participants’ baseline characteristics are indicated in Table 1. Complete data were available for 123 patients, and these were considered in this analysis. Mean age was 67.8 years, and the mean number of exacerbations in these patients’ history was 3.1/year (Table 1).

**Table 1:** Patient characteristics

	All participants N = 127	Selected N = 69
Age at enrolment (years) mean ± SD	66.8 ± 8.6	66.3 ± 8.8
Female sex, *n* (%)	59 (46.5%)	25 (36.2%)
Smoking history (pack-years), median (IQR)	47.0 (33.7–60.0)	NA
Medication for COPD, *n* (%)	127 (100%)	NA
Influenza vaccination during previous year, *n* (%)	114 (89.8%)	NA
Pneumococcal vaccination during previous year, *n* (%)	12 (9.4%)	NA
COPD severity status at enrolment, *n* (%)		
Mild	0 (0%)	0 (0%)
Moderate	57 (44.9%)	34 (49.3%)
Severe	51 (40.2%)	27 (39.1%)
Very severe	19 (15.0%)	8 (11.6%)
Number of patients reporting exacerbations in preceding 12 months, *n* (%)		
One exacerbation	28 (22.0%)	14 (20.3%)
Two exacerbations	37 (29.1%)	16 (23.2%)
Three exacerbations	25 (19.7%)	16 (23.2%)
Four or more exacerbations	37 (29.1%)	23 (33.3%)
Number of exacerbations in preceding 12 months, mean ± SD and median (IQR)	3.1 ± 2.3 and 2 (2–4)	3.5 ± 2.7 and 3 (2–5)

Patient characteristics from [12]

*COPD* chronic obstructive pulmonary disease, *IQR* interquartile range, *n* number, *NA* non-available, *SD* standard deviation

For this study, we selected all patients (in total 65) with NTHi positive samples from both ≥ 1 ST and ≥ 1 EX and all patients (in total 22) with Mcat positive samples from both ≥ 1 ST and ≥ 1 EX. The two lists partially overlap; therefore, the total number of patients investigated was 69, and the total number of samples was 174. Considering the COPD severity status, this subset of subjects showed similar patient characteristics (differences < 6%) as the enrolled cohort (Table 1). The ST/EX sample pairs of each subject were chosen randomly and on average there was a median interval between the two samples of about 161 days (with first and third quartiles of 78 days and 315 days, respectively).

### NTHi and Mcat vaccine antigens are expressed in the lung

To determine whether the NTHi and Mcat vaccine antigens are expressed in the lungs, RNA from sputum samples was analysed using RT-ddPCR. NTHi genes were tested in all samples that were positive for NTHi by PCR. Expression of the genes was detectable in all these samples and showed gene-specific expression levels (Additional file 1: Fig. S1). Mcat genes *uspA2*, *parE*, and *polA* were tested in all samples that were PCR positive for Mcat. Expression of *uspA2* and *polA* was detectable in all these samples, while *parE* expression was only detectable in a limited number of samples (Additional file 1: Fig. S2).

### Expression of NTHi genes was similar between ST and EX

To determine whether the expression of NTHi vaccine antigens is different between ST and EX, gene expression was determined in sputum samples of 65 patients who had NTHi positive samples during both ST and EX. Expression of NTHi genes *pd*, *pe*, *pilA*, *gapA*, and *ompP6* was determined by RT-ddPCR. Of the housekeeping genes, median expression of *ompP6* was highest. Of the NTHi-Mcat candidate genes, median expression of *pd* was highest, followed by *pe*, and *pilA* was lowest (Table 2). Absolute RNA concentrations were compared using both group and paired (by patient) statistics and no significant differences were found between ST and EX (all p > 0.05) (Additional file 1: Fig. S1).

**Table 2:** Gene expression of NTHi and Mcat genes in sputum samples

Gene (visit)	Samples, *n*	Median, copies/μl	IQR, copies/μl
NTHi genes in NTHi positive samples			
*gapA* (ST)	55	6.14	0.37–53.12
*gapA* (EX)	47	23.10	0.72–135.7
*ompP6* (ST)	58	0.87	0.24–11.28
*ompP6* (EX)	50	0.93	0.23–15.97
*pd* (ST)	24	0.06	0.06–2.35
*pd* (EX)	32	0.06	0.06–1.86
*pe* (ST)	52	2.00	0.11–42.58
*pe* (EX)	53	4.43	0.11–125.8
*pilA* (ST)	55	88.90	2.82–527.9
*pilA* (EX)	48	43.11	0.23–789.7
Mcat genes in Mcat positive samples			
*uspA2* (ST)	17	4.71	3.68–24.98
*uspA2* (EX)	18	4.03	3.22–128.8
*parE* (ST)	3	1.34	0.11–39.84
*parE* (EX)	4	0.44	0.11–1.05
*polA* (ST)	15	0.33	0.22–1.01
*polA* (EX)	14	0.42	0.24–1.09

*EX* exacerbation visit, *IQR* interquartile range,* n* number, *ST* stable visit

**Fig. 1:**
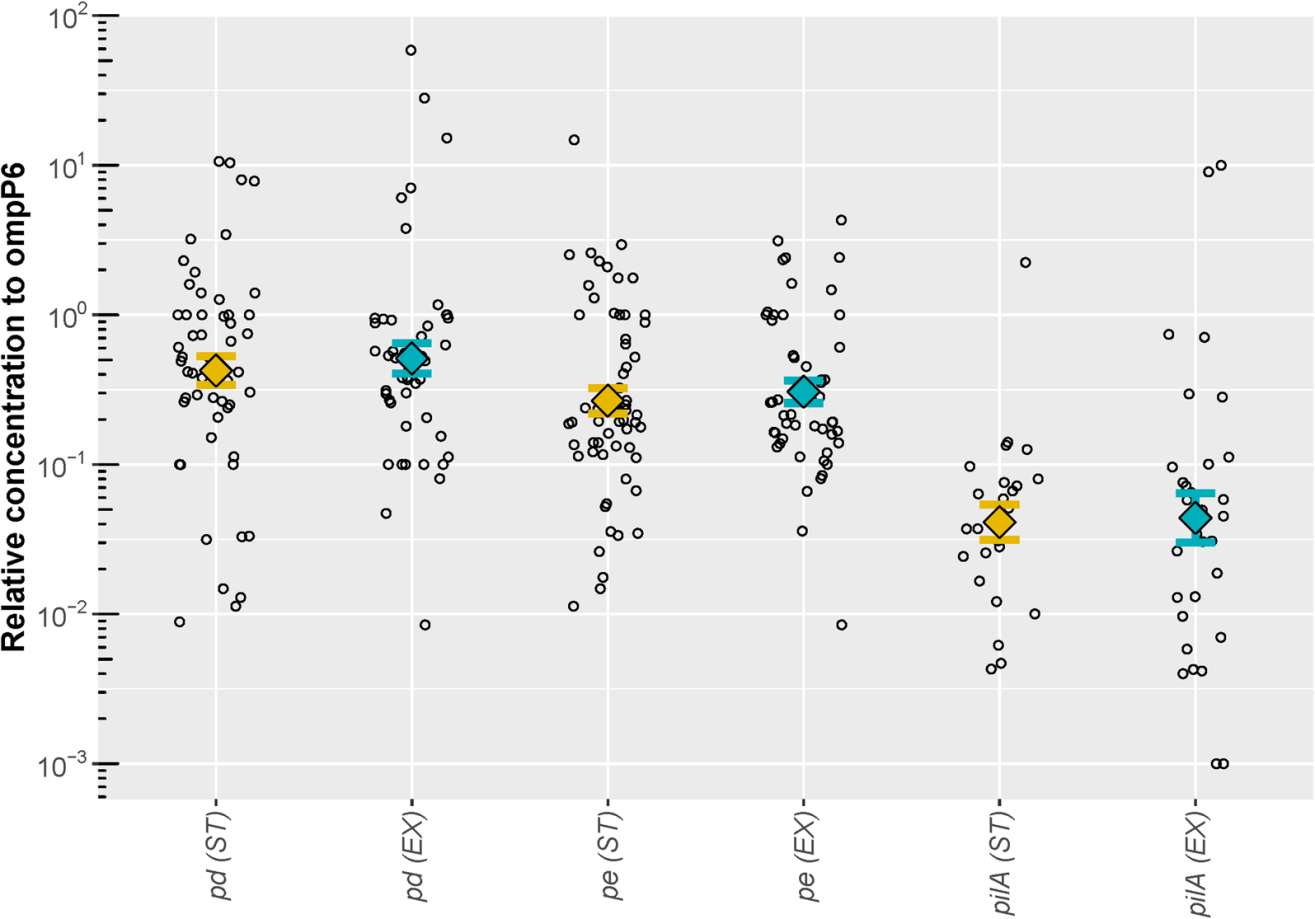
Relative RNA concentration of NTHi genes in NTHi positive samples, normalised to ompP6. Relative RNA concentration of the NTHi genes *pd*, *pe*, and *pilA*, in copies per μl, normalised to the housekeeping gene *ompP6* RNA*.* Relative expressions were not significantly different between ST and EX with either paired t-tests or Welch’s two-sample t-tests. *ST* stable visit, *EX* exacerbation visit. Each circle represents normalised gene concentration observed in a single sample. Geometric mean relative concentration of each antigen is represented by the colored diamond symbols (yellow for ST and blue for EX samples) with error bars showing standard error widths

**Fig. 2:**
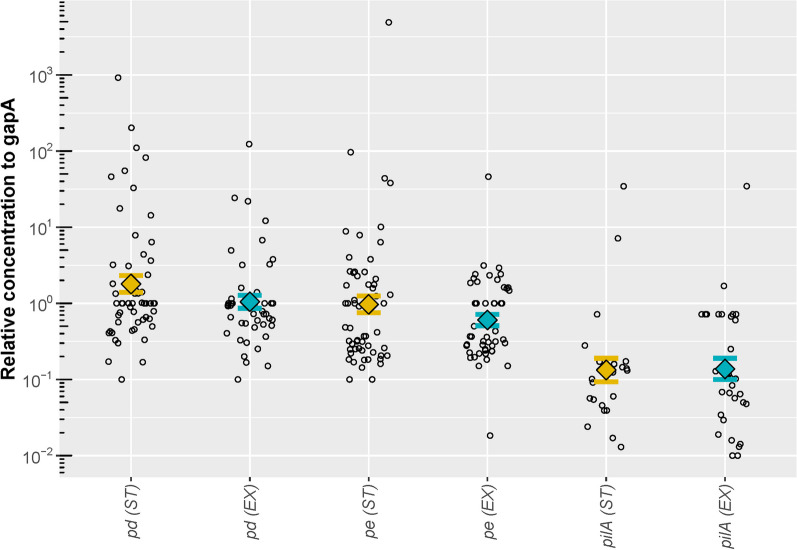
Relative RNA concentration of NTHi genes in NTHi positive samples, normalised to gapA. Relative RNA concentration of the NTHi genes *pd*, *pe*, and *pilA*, in copies per μl, normalised to the housekeeping gene *gapA* RNA*.* Relative expressions were not significantly different between ST and EX with either paired t-tests or Welch’s two-sample t-tests. *ST* stable visit, *EX* exacerbation visit. Each circle represents normalised gene concentration observed in a single sample. Geometric mean relative concentration of each antigen is represented by the colored diamond symbols (yellow for ST and blue for EX samples) with error bars showing standard error widths

RNA concentration of the NTHi specific genes *pd*, *pe*, and *pilA* were subsequently analysed relative to housekeeping genes *ompP6* (Fig. 1) and *gapA* (Fig. 2) or to their average combination. Both group and paired statistics confirmed there were no significant differences in the relative concentrations between ST and EX (all p > 0.05) when normalized to *ompP6* (Fig. 1), to *gapA* (Fig. 2), or to their average combination (data not shown).

### Expression of Mcat uspA2 was similar between ST and EX

To determine whether expression of the Mcat *uspA2* gene is different between ST and EX, gene expression was determined in sputum samples of 22 people who had Mcat positive samples during both ST and EX. Expression of Mcat gene *uspA2* and housekeeping genes *polA* and *parE* was determined using RT-ddPCR (Table 2). Absolute RNA concentrations were compared with both group and paired (by patient) statistics and were not significantly different between ST and EX (all p > 0.05) (Additional file 1: Fig. S2). Too few ST samples (n = 3) and EX samples (n = 4) had detectable expression of *parE*, and these were not from the same patients, so *parE* was excluded from further analysis.

**Fig. 3:**
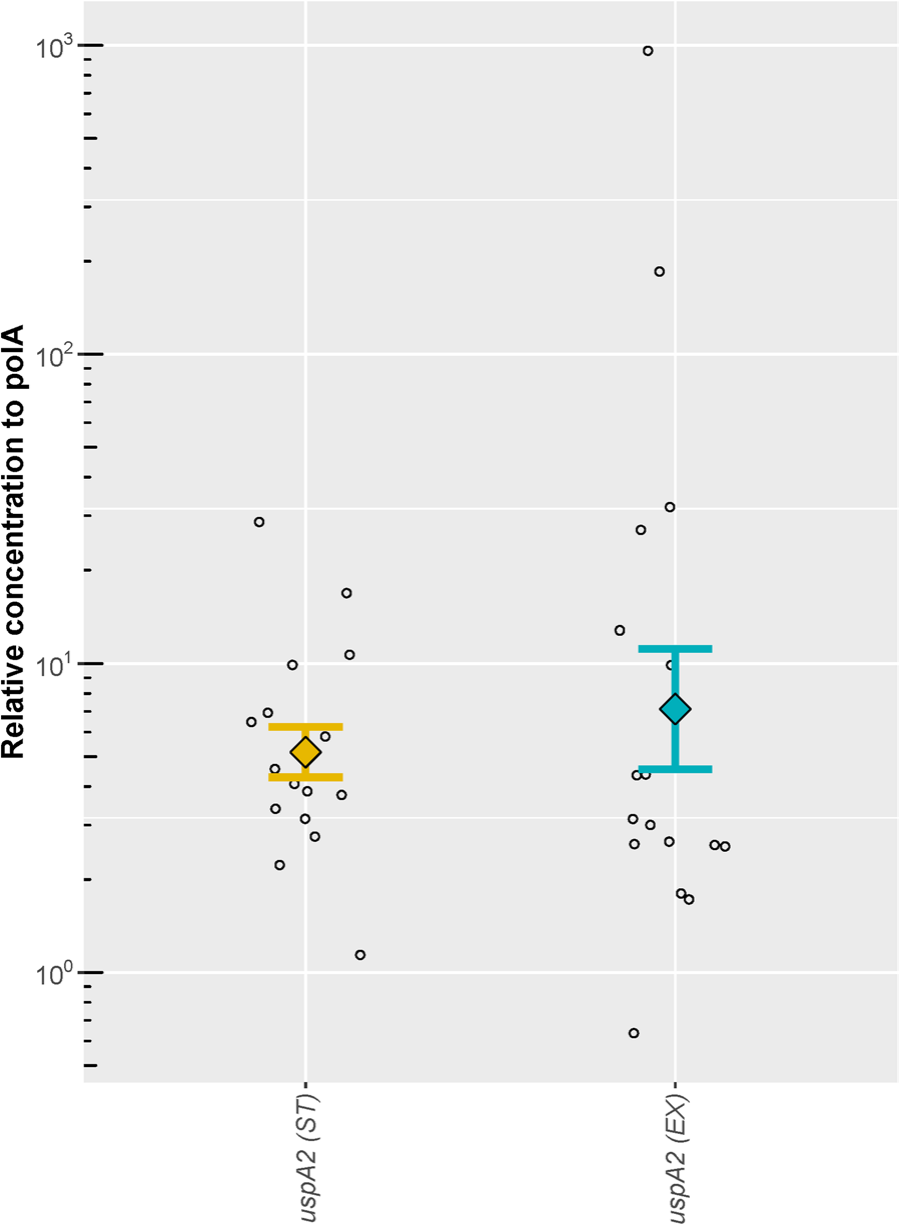
Relative RNA concentration of Mcat genes in Mcat positive samples. Relative RNA concentration of the Mcat gene *uspA2*, in copies per μl, normalised to the housekeeping gene *polA* RNA*.* Relative expressions were not significantly different between ST and EX with either paired t-tests or Welch’s two-sample t-tests. *ST* stable visit, *EX* exacerbation visit. Each circle represents normalised gene concentration observed in a single sample. Geometric mean relative concentration of each antigen is represented by the colored diamond symbols (yellow for ST and blue for EX samples) with error bars showing standard error widths

The *uspA2* RNA concentration was subsequently determined relative to housekeeping gene *polA* in each patient. Both group and paired statistics confirmed that there were no significant differences between *uspA2* relative expression levels at the ST and EX (p > 0.05) (Fig. 3).

### pd, pilA and uspA2 gene expression is weakly associated with COPD history of exacerbations

Antigen expression overall, or separating ST and EX was analysed for correlations with: age; gender; COPD severity at enrolment or at each single visit; total number of exacerbation events during the study and the year before enrolment; NTHi and Mcat DNA PCR concentrations measured at single visits or as average of all visits. Very few statistically relevant correlations were observed by linear regression modeling. Only *pd* (p ≤ 10^–15^) NTHi gene showed a correlation of absolute concentration with average DNA PCR quantification for NTHi, with a difference between ST and EX. A similar trend was observed for *ompP6* and *gapA* housekeeping genes and in fact this correlation was not statistically relevant if antigens concentrations were normalised to the measured concentrations of housekeeping genes. Moreover, *pd* (p = 0.019) and *pilA* (p < 10^–15^) absolute expression correlated with the number of exacerbations in the year before the enrolment, showing a difference between ST and EX. However, these correlations were not statistically relevant if antigen concentrations were normalised. Another difference was observed for the *uspA2* relative concentration to the *polA* housekeeping gene when samples were analysed by COPD severity at enrolment (p = 0.022), and more importantly when we combined the severity status at enrolment with current visit status (EX or ST) (p = 0.013). The expression of the *pd*, *pe*, *pilA* and *uspA2* genes was not correlated with the total number of exacerbations presented during the study, or to the DNA PCR quantification for NTHi and Mcat (on average or at each single visit).

## Discussion

In a prospective, observational cohort study we analysed expression of NTHi-Mcat vaccine candidate genes in 174 sputum samples from 69 COPD patients positive for the presence of NTHi and/or Mcat bacteria. We selected patients that had sputum samples positive for the bacterial pathogens during at least one ST and one EX. We showed for the first time that the expression of almost every NTHi-Mcat vaccine candidate gene was detectable in sputum samples and that expression was detectable during both ST and EX. In addition, we found that there was no difference in expression of these genes between ST and EX. Finally, we observed a slight association of *pd* and *pilA* gene expression with the number of AECOPD events before enrolment that is completely undetectable when antigen expression is normalized to the housekeeping genes expression level.

Normalization of gene expression data against the expression of multiple household genes has been recommended to limit errors and increase the accuracy of results essential for a good comparison of gene expression from sputum samples [15]. Therefore, in addition to normalization of NTHi vaccine genes against *ompP6* and *gapA* individually*,* we also performed normalization to the average combination of these two household genes using the method proposed by Riedel et al. [14]. The results were not different from the data normalized against the single household genes.

NTHi and Mcat are often present during exacerbation states and these pathogens are therefore thought to play a role in triggering exacerbations [8]. In this AERIS study, the presence of NTHi and Mcat, but not of other bacteria, was associated with a heightened exacerbation risk [12], therefore we wanted to investigate whether this association is perhaps due to a modulation of expression of NTHi and Mcat virulence factors during exacerbations compared to stable state.

In a similar study in Spain that was performed from October 2009 to October 2010, *Pseudomonas aeruginosa* and *Streptococcus pneumoniae* were isolated most frequently from AECOPD sputum while NTHi and Mcat were isolated as well [16], indicating pathogens may vary by year and/or geographic location.

It was previously concluded, based on the presence of the pathogens in the sputum, that NTHi and Mcat detection was associated with AECOPD occurrence [12]. The current study confirms this based on the continued expression of key virulence genes during the ST and EX. In addition, because the expression of the housekeeping genes was not different between ST and EX, it appears that the pathogens are not increasing or modulating the quantity of the transcripts of these genes during the exacerbations.

In the AERIS study, analysing the lung microbiome in AECOPD, the relative abundance of *Moraxella* was found to be increased in samples taken during exacerbations compared to those taken during ST [17]. Indeed, in a previous study, the lung microbiome was found to be dynamic, microbiome changes were associated with exacerbations, and the relative abundance of *Moraxella* was overall increased, although there was some heterogeneity, as in some patients *Moraxella* abundance was decreased at exacerbation [18]. In that same study, it was found that treatments potentially alter the lung microbiome. In particular, a reduction in microbial diversity and an increased Proteobacteria:Firmicutes ratio were observed in patients treated with steroids alone, whereas the trend was reversed in patients treated with antibiotics [18]. In this previous study, the technique used to determine the relative abundance of bacteria (16s rRNA gene sequencing) did not allow identification at the species level, only at the genus level [17, 18], so the changes they found may not be relevant for specific individual species. For the current analyses, we selected ST and EX samples from the same patients who were Mcat or NTHi positive, which is different from the two previous studies, where any samples from ST and EX were analysed regardless of whether they contained the pathogen or not [17, 18]. Relative abundance of *Moraxella* might have been similar in those studies as well if ST and EX samples had been used that were matched for being Mcat positive.

In two small, earlier studies of the lung microbiome, overall microbial diversity was less in severe or very severe patients than in mild-to-moderate or moderate-to-severe COPD patients [19, 20]. We found that absolute expression of the Mcat housekeeping gene *polA*, and absolute and relative expressions of the Mcat gene *uspA2*, were not different between ST and EX. These results may indicate that if the total lung microbiome diversity during exacerbations was decreased, while Mcat remained present in the same amount, then we have an increase of the relative amount of Mcat.

One limitation of this study was the limited sample size analysed in addition to the non-consideration of the impact of treatment on the NTHi and Mcat population. Another limitation is that we were not able to analyse gene expression in patients with AECOPD for which no sputum samples were available at stable visits.

A strength of the study is the application of RT-ddPCR, which is an ultrasensitive method for RNA quantification. This method allows for monitoring targets in complex backgrounds such as detecting mRNA from individual bacterial genes in samples that also contain human material and material from various other microbes.

## Conclusions

The four NTHi-Mcat vaccine candidate NTHi and Mcat genes were expressed in ex-vivo sputum samples from COPD patients during both ST and EX, and the expression levels appeared to be gene-specific. No differences in gene expression were detectable between ST and EX, suggesting that the presence of these pathogens is not limited to the exacerbations, but rather that these pathogens colonised the patients’ lungs. The presence of mRNA from the NTHi-Mcat vaccine candidate genes in the sputum of COPD patients suggests that these antigens are expressed in the lung and illustrates the relevance of the inclusion of these candidates in a NTHi-Mcat vaccine.

### Supplementary Information

**Additional file 1: Table S1.** PCR primer sequences for NTHi and Mcat genes. **Figure S1.** NTHi gene RNA concentrations in NTHi positive samples did not differ between stable visits (ST) and exacerbation visits (EX). **Figure S2.** Mcat gene RNA concentrations in Mcat positive samples did not differ between stable visits (ST) and exacerbation visits (EX). Additional methods.

## Data Availability

Anonymised individual participant data and study documents can be requested for further research from https://www.clinicalstudydatarequest.com.

## Abbreviations

AECOPD: Acute Exacerbation of Chronic Obstructive Pulmonary Disease
AERIS: Acute Exacerbation and Respiratory InfectionS
COPD: Chronic Obstructive Pulmonary Disease
ddPCR: Droplet digital PCR
EX: Exacerbation visit
FEV1: Forced Expiratory Volume in 1 s
*gapA*: Glyceraldehyde 3-phosphate dehydrogenase gene
Gapdh: Glyceraldehyde 3-phosphate dehydrogenase
Mcat: *Moraxella catarrhalis*
NTHi: Non-typeable *Haemophilus** influenzae*
OmpP6: Outer membrane protein P6
*ompP6*: Outer membrane protein P6 gene
ParE: DNA topoisomerase IV subunit B
*parE*: DNA topoisomerase IV subunit B gene
PD: Protein D
*pd*: Protein D gene
PE: Protein E
*pe*: Protein E gene
PilA: Type IV pilin subunit protein
*pilA*: Type IV pilin subunit protein gene
PolA: DNA polymerase I
*polA*: DNA polymerase I gene
ST: Stable routine visit
UspA2: Universal stress protein A homolog 2
*uspA2*: Universal stress protein A homolog 2 gene
