# Correction to: Ex-vivo RNA expression analysis of vaccine candidate genes in COPD sputum samples

**DOI:** 10.1186/s12931-024-02826-x

**Published:** 2024-05-29

**Authors:** Cecilia Brettoni, Alessandro Muzzi, Simona Rondini, Vincent Weynants, Silvia Rossi Paccani

**Affiliations:** 1 GSK, Via Fiorentina 1, Siena, Italy; 2 GSK, Rixensart, Belgium

**Correction to: Respiratory Research (2023) 24:243**

10.1186/s12931-023-02525-z

Following publication of the original article [1], the author identified the errors in tables and supplementary material. The corrections have been listed below:

In Result section under “Patients and samples”, the mean age was incorrectly given as “67.8 years” and should have be “66.8 years”. The correct sentence should read “Mean age was 66.8 years, and the mean number of exacerbations, in these patients’ history was 3.1/year (Table 1)”.

In Table 2, some values were inadvertently mismatched during the copy-pasting process from the source data table. This mistake has no impact on the conclusion of the manuscript. This has been corrected with this erratum.

In Supplementary Table 1, some primers sequences were also erroneously duplicated. This has been updated now.

**Table 2:** Gene expression of NTHi and Mcat genes in sputum samples

Gene (visit)	Samples, *n*	Median, copies/µl	IQR, copies/µl
NTHi genes in NTHi positive samples			
*gapA* (ST)	52	2.00	0.11–40.40
*gapA* (EX)	53	4.43	0.11–117.66
*ompP6* (ST)	55	88.90	4.81–492.77
*ompP6* (EX)	48	43.11	0.48–706.50
*pd* (ST)	55	6.14	0.50–48.04
*pd* (EX)	47	23.10	0.87–109.46
*pe* (ST)	58	0.87	0.26–10.89
*pe* (EX)	50	0.93	0.24–14.55
*pilA* (ST)	24	0.06	0.06–1.69
*pilA* (EX)	32	0.06	0.06–1.84
Mcat genes in Mcat positive samples			
*uspA2* (ST)	17	4.71	3.81–21.31
*uspA2* (EX)	18	4.03	3.29–90.55
*parE* (ST)	3	1.34	0.73–20.59
*parE* (EX)	4	0.44	0.12–0.86
*polA* (ST)	15	0.33	0.22–0.89
*polA* (EX)	14	0.42	0.26–0.97

EX, exacerbation visit; IQR, interquartile range; *n*, number; ST, stable visit

### Electronic supplementary material

Below is the link to the electronic supplementary material.

Supplementary Material 1
