# Supplementary material for: Ex-vivo RNA expression analysis of vaccine candidate genes in COPD sputum samples
Source: Respir Res. 2023 Oct 5;24:243. doi: 10.1186/s12931-023-02525-z (PMC10552247; doi:10.1186/s12931-023-02525-z)
Supplement: Supplementary file 1 — Additional file 1: Table S1. PCR primer sequences for NTHi and Mcat genes. Figure S1. NTHi gene RNA concentrations in NTHi positive samples did not differ between stable visits (ST) and exacerbation visits (EX). Figure S2. Mcat gene RNA concentrations in Mcat positive samples did not differ between stable visits (ST) and exacerbation visits (EX). Additional methods. [file 12931_2023_2525_MOESM1_ESM.docx]

# Additional file 1

## ***Table S1 PCR primer sequences for NTHi and Mcat genes***

| **Target gene** | **Primer names and sequences (5′ to 3′)** | **Ta (ºC)** |
| --- | --- | --- |
| *gapA* | GAPDH_625_F: ACAGGTGCRGCGAAAGCACAGGTGCRGCGAAAGC  GAPDH_703_R: TTGGAACACGGAAAGCCATAC  GAPDH_657_MGB probe: CCTGCATTAAACGGTAAAACCTGCATTAAACGGTAAA | 54 |
| *ompP6* | OmpP6_F: GCAGATGCAGTTAAAGGTTAGCAGATGCAGTTAAAGGTTA  OmpP6_R: TTCTTCACCGTAAGATACTG  OmpP6_MGB probe: AGGTGTTGATGCTGGTAA | 54 |
| *pd* | pD_202_F: CAAGATTTAGCAATGACTAARGATGGT  pD_298_R: GACGATGTGGGAATTTTTTYG  pD_MGB probe: TGGTTATTCACGATCACTT | 55 |
| *pe* | pE_213_F: GGTGAATTTAGATAAGGGATTGTATGTTTGGTGAATTTAGATAAGGGATTGTATGTTT  pE_284_R: TTATACTGACGAACAGAACGTGCAT  pE_243_MGB probe: TCCTGAGCCTAAACGTTCCTGAGCCTAAACGT | 54 |
| *pilA* | PilA_43b_F: TTAATMGARCTAATGATTGTGATTGCA  PilA_155_R: GAHGCTTGCAGTAATTCRGATACC  PilA_MGB probe: CTATTTTAGCCACTATCGC | 55 |
| *uspA2* | UspA2_811_F: GCCCAAGCTGCCCTAAGTG  UspA2_812_R: GCGGTCGCATTAAACTTACCA  UspA2_MGB probe: TCTATTCCAGCCTTATAGCG | 54 |
| *polA* | polA­_F: GAGCGTGCAGCCATTAGAGCGTGCAGCCATTA  polA_R: CGCAATCATGGCAAGTT  polA_MGB probe: ACAAGGCTCAGCTGCACAAGGCTCAGCTGC | 54 |
| *parE* | ParE_823_F: CAAGGTGGCACGCATGTC  ParE_824_R: AAACTCACGCAGTGCCTCAA  ParE_MGB probe: CACCTGTGCGTAAACCAT | 54 |

F, forward primer; R, reverse primer; Ta, annealing temperature; MGB probe, minor groove binder probe

## ***Figure S1 NTHi gene RNA concentrations in NTHi positive samples did not differ between stable visits (ST) and exacerbation visits (EX)***


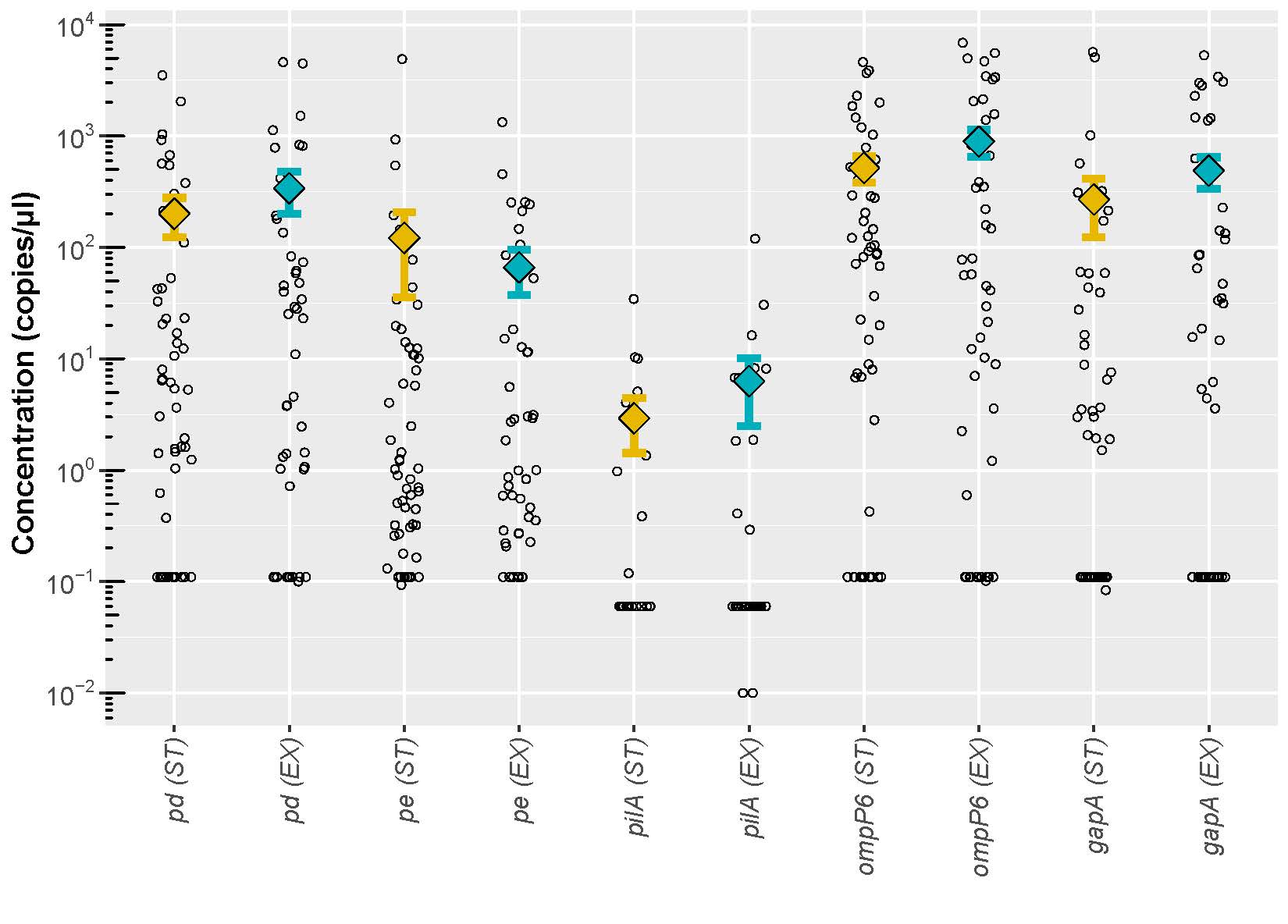


RNA concentration of the NTHi genes *pd*, *pe*, *pilA*, *gapA*, and *ompP6* in copies per μl. The concentrations were not significantly different between ST and EX with either paired t-tests or Welch’s two-sample t-tests.

Each circle represents a gene’s RNA concentration observed in a single sample. The mean RNA concentration of each gene is indicated by the colored diamond symbols (yellow for ST and blue for EX samples) with whiskers representing the standard errors.

## ***Figure S2 Mcat gene RNA concentrations in Mcat positive samples did not differ between stable visits (ST) and exacerbation visits (EX)***


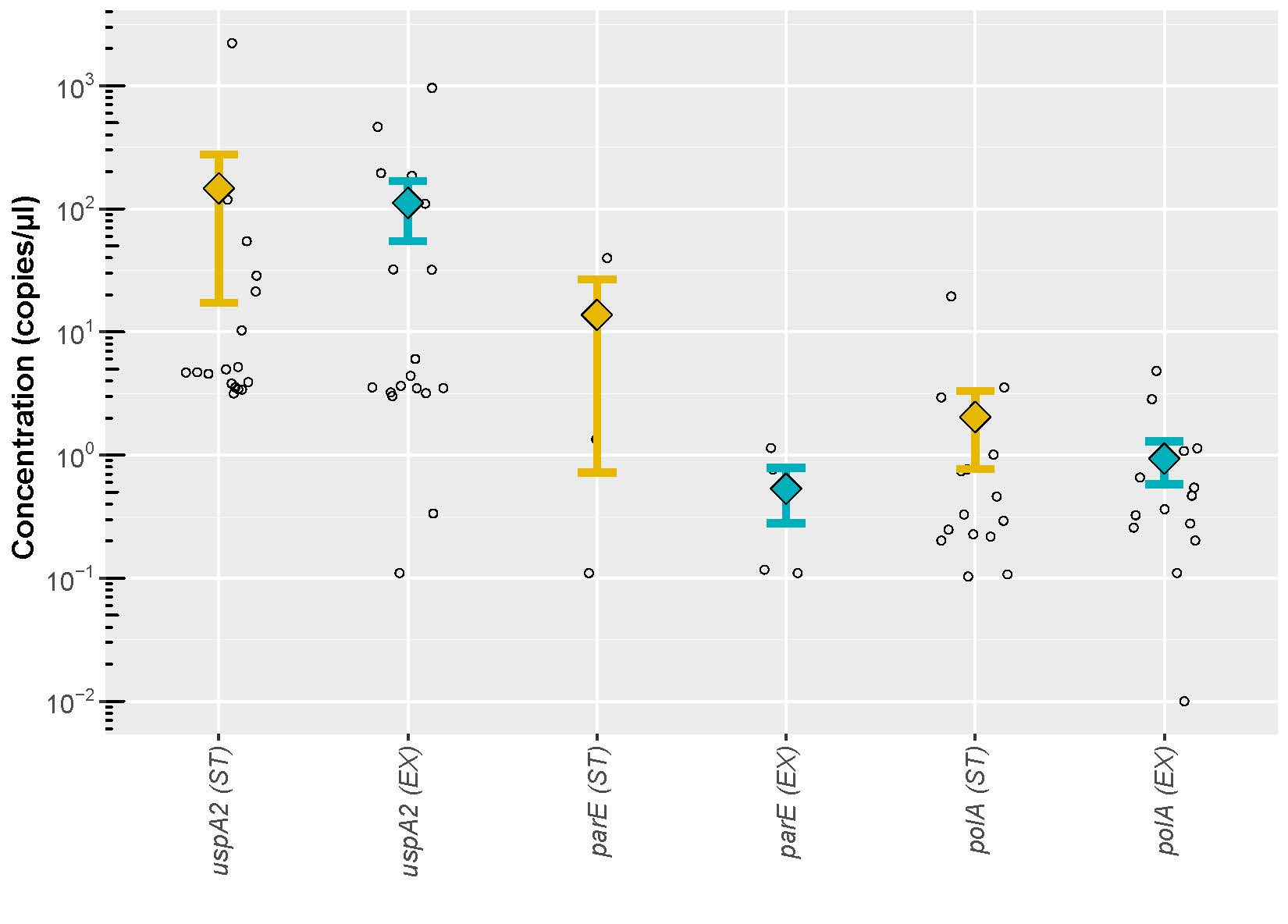


RNA concentration of the Mcat genes *uspA2*, *parE*, and *polA* in copies per μl. The concentrations were not significantly different between ST and EX with either paired t-tests or Welch’s two-sample t-tests.

Each circle represents a gene’s RNA concentration observed in a single sample. The mean concentration of each gene is indicated by the colored diamond symbols (yellow for ST and blue for EX samples) with whiskers representing the standard errors.

# Additional Methods

## ***Regression models***

We have generated various regression models to investigate whether some of the subjects’ features and parameters, such as COPD status at enrolment or at a subsequent visit, were correlated with the RNA concentrations of the antigens as measured by reverse-transcriptase droplet digital PCR (RT-ddPCR).

We used the following rank linear regression models (implemented by *rfit* function of R Rfit package) to inspect correlations between antigens’ RNA concentration (absolute and log-normalised to the selected housekeeping genes expression in the same sample, at stable visits and exacerbations) with key features observed in the study. In case of relationships with discrete categorical variables, we applied a rank-based ANOVA model (implemented by *raov* function of R Rfit package).

1. *Relationship with total number of exacerbations during the study*

(number of exacerbations during the study) ~ (Antigen RNA concentration)

1. *Relationship with the average quantification of Hi or Mcat pathogens DNA*

(subject’s average amount of pathogen DNA) ~ (Antigen RNA concentration)

1. *Relationship with the COPD status at enrolment (airflow limitation: Moderate, Severe, or Very severe)*

(Antigen RNA concentration) ~ (airflow limitation at enrolment)

1. *Relationship with the COPD status at each visit (airflow limitation: Missing confirmed, Moderate, Severe, or Very severe)*

(Antigen RNA concentration) ~ (airflow limitation at single visit)

1. *Relationship with the subject’s gender (Male or Female)*

(Antigen RNA concentration) ~ (gender)

1. *Relationship with the subject’s age*

(Antigen RNA concentration) ~ (age)

1. *Relationship with the number of exacerbations in the year before enrolment*

(Antigen RNA concentration) ~ (number of exacerbations in the year before enrolment)

## ***RNA quantity determination and RNA amounts used in the experiments***

The quantity and quality of the RNA isolated was determined in ten sputum samples that were used for the setup experiments. RNA quantity was assessed with a 2100 Bioanalyzer System (Agilent Technologies). The RNA concentrations ranged from 1 to 51 ng/μl (mean 10.6 ng/μl) and the RNA integrity numbers (RIN) ranged from N/A to 5.20. Note that the RIN cannot be determined (N/A) using the Prokaryote Total RNA Nano assay when the RNA concentration is very low.

RT-ddPCRs were performed using 1 μl and/or serial dilutions of each RNA preparation. The quantity of RNA used in the RT-ddPCR varied on the basis of the expression level of each gene in order to avoid that the RT-ddPCR reached the saturation point where the RNA measurements would be impaired.
