# Supplementary material for: Correction to: Ex-vivo RNA expression analysis of vaccine candidate genes in COPD sputum samples
Source: Respir Res. 2024 May 29;25:225. doi: 10.1186/s12931-024-02826-x (PMC11137875; doi:10.1186/s12931-024-02826-x)
Supplement: Supplementary file 1 — Supplementary Material 1 [file 12931_2024_2826_MOESM1_ESM.docx]

# Additional files

**Table S1 PCR primer sequences for NTHi and Mcat genes**

| **Target gene** | **Primer names and sequences (5′ to 3′)** | **Ta (ºC)** |
| --- | --- | --- |
| *gapA* | GAPDH_625_F: ACAGGTGCRGCGAAAGC  GAPDH_703_R: TTGGAACACGGAAAGCCATAC  GAPDH_657_MGB probe: CCTGCATTAAACGGTAAA | 54 |
| *ompP6* | OmpP6_F: GCAGATGCAGTTAAAGGTTA  OmpP6_R: TTCTTCACCGTAAGATACTG  OmpP6_MGB probe: AGGTGTTGATGCTGGTAA | 54 |
| *pd* | pD_202_F: CAAGATTTAGCAATGACTAARGATGGT  pD_298_R: GACGATGTGGGAATTTTTTYG  pD_MGB probe: TGGTTATTCACGATCACTT | 55 |
| *pe* | pE_213_F: GGTGAATTTAGATAAGGGATTGTATGTTT  pE_284_R: TTATACTGACGAACAGAACGTGCAT  pE_243_MGB probe: TCCTGAGCCTAAACGT | 54 |
| *pilA* | PilA_43b_F: TTAATMGARCTAATGATTGTGATTGCA  PilA_155_R: GAHGCTTGCAGTAATTCRGATACC  PilA_MGB probe: CTATTTTAGCCACTATCGC | 55 |
| *uspA2* | UspA2_811_F: GCCCAAGCTGCCCTAAGTG  UspA2_812_R: GCGGTCGCATTAAACTTACCA  UspA2_MGB probe: TCTATTCCAGCCTTATAGCG | 54 |
| *polA* | polA­_F: GAGCGTGCAGCCATTA  polA_R: CGCAATCATGGCAAGTT  polA_MGB probe: ACAAGGCTCAGCTGC | 54 |
| *parE* | ParE_823_F: CAAGGTGGCACGCATGTC  ParE_824_R: AAACTCACGCAGTGCCTCAA  ParE_MGB probe: CACCTGTGCGTAAACCAT | 54 |

F, forward primer; Mcat, *Moraxella catarrhalis*; MGB probe, minor groove binder probe; NTHi, non-typeable *Haemophilus influenzae*; PCR, polymerase chain reaction; R, reverse primer; Ta, annealing temperature;
